# Inhibition of Fungal Growth and Aflatoxin B_1_ Synthesis in *Aspergillus flavus* by Plasma-Activated Water

**DOI:** 10.3390/foods12132490

**Published:** 2023-06-26

**Authors:** Qihuan Yao, Hangbo Xu, Jie Zhuang, Dongjie Cui, Ruonan Ma, Zhen Jiao

**Affiliations:** 1 Zhengzhou Research Base, State Key Laboratory of Cotton Biology, School of Agricultural Sciences, Zhengzhou University, Zhengzhou 450001, Chinajiaozhen@zzu.edu.cn (Z.J.); 2 Henan Key Laboratory of Ion-Beam Bioengineering, School of Agricultural Sciences, Zhengzhou University, Zhengzhou 450052, China; 3 Sanya Institute, Zhengzhou University, Zhengzhou 450001, China; 4 Suzhou Institute of Biomedical Engineering and Technology, Chinese Academy of Sciences, Suzhou 215163, China

**Keywords:** plasma-activated water, *Aspergillus flavus*, peroxynitrite, fungicidal effect, inhibitory effect

## Abstract

The gaseous reactive oxygen/nitrogen species (RONS) generated by cold atmospheric plasma (CAP) can effectively inactivate *Aspergillus flavus (A. flavus)* and prolong the shelf-life of food. Plasma-activated water (PAW) is the extension of cold plasma sterilization technology. Without the limitation of a plasma device, PAW can be applied to more scenarios of food decontamination. However, the efficacy of PAW as a carrier of RONS for eradicating *A. flavus* or inhibiting its growth remains unclear. In this study, the immediate fungicidal effect and long-term inhibitory effect of PAW on *A. flavus* were investigated. The results demonstrated that 60-min instant-prepared PAW could achieve a 3.22 log reduction CFU/mL of *A. flavus* and the fungicidal efficacy of PAW gradually declined with the extension of storage time. Peroxynitrite (ONOO^−^/ONOOH) played a crucial role in this inactivation process, which could damage the cell wall and membrane structure, disrupt intracellular redox homeostasis, and impair mitochondrial function, ultimately leading to fungal inactivation. In addition to the fungicidal effect, PAW also exhibited fungistatic properties and inhibited the synthesis of aflatoxin B_1_ (AFB_1_) in *A. flavus*. By analyzing the cellular antioxidant capacity, energy metabolism, and key gene expression in the AFB_1_ synthesis pathway, it was discovered that PAW can significantly reduce ATP levels, while increasing SOD and CAT activity during 5-d cultivation. Meanwhile, PAW effectively suppressed the expression of genes related to AFB_1_ synthesis.

## 1. Introduction

*Aspergillus flavus* (*A. flavus*) is a soil-borne saprophytic fungus that infects and contaminates crops [1]. *A. flavus* produces aflatoxins, which are highly toxic and pose serious threats to food quality, safety, and human health [2]. It has been reported that about 25% of global food crops worldwide are contaminated with aflatoxins [3]. Therefore, the decontamination of *A. flavus* and aflatoxins are critical issues that need to be addressed [4].

Cold atmospheric plasma (CAP) attracts increasing attention as a novel non-thermal technology for food decontamination and preservation [5]. CAP is generated by electrical discharge, which refers to ionized gas that processes a variety of reactive oxygen/nitrogen species (RONS). These RONS have been proven to be the major agents in microbial inactivation [6,7]. Numerous investigations have also confirmed the antifungal effects of CAP against *A. flavus.* For instance, Dasan et al. demonstrated that CAP could compromise the integrity of *A. flavus* spore cells by scanning electron microscopy (SEM) observations [8,9]. The decontamination efficacy was found to increase with the increasing applied reference voltage and frequency. Upon further study, Dasan et al. discovered that air plasma was more effective than nitrogen plasma in eliminating *A. flavus* spores due to the formation of ROS in the presence of oxygen [10]. Devi et al. found that a 60-W and 15-min CAP treatment could effectively inactivate 99.5% of *A. flavus*, and significantly reduce the production of AFB_1_ by 96.8% [11]. Mošovská and Ott et al. employed optical emission and absorption spectroscopy to confirm the production of gaseous RONS, e.g., hydroxyl radical (·OH), ozone (O_3_), nitrogen oxides (NO_x_). These RONS induce the inactivation of *A. flavus* [2,12]. Recently, Lin et al. employed a novel rotary plasma jet to inhibit *A. flavus* and found CAP effectively inhibited the growth of *A. flavus* on peanuts. Moreover, the content of AFB_1_ was significantly reduced. Aflatoxin concentrations in CAP treated (200 W and 5 min) and untreated peanuts were 16.5 and 363.7 ppb after 29-d storage, respectively [3].

These aforementioned research articles demonstrated the decontamination ability of CAP against *A. flavus* and Aflatoxin. However, CAP decontamination faces plenty of problems in practical application. For example, the samples with irregular shapes cannot be treated homogeneously by CAP [13] and the large-scale processing is difficult to achieve [14]. Moreover, the UV and electromagnetic fields in CAP may pose potential hazards to the operators and damage delicate biological materials [15]. Thus, to successfully transfer CAP decontamination into the practical application, a novel CAP-based technology should be proposed. Recently, plasma-activated water (PAW) has been born to meet the need. PAW is generated by CAP treatment of water, which also possesses rich RONS (such as ∙OH, superoxide anion (∙O_2_^−^), singlet oxygen (^1^O_2_), hydrogen peroxide (H_2_O_2_), nitrate (NO_3_^−^), nitrite (NO_2_^−^), and peroxynitrite (ONOO^−^/ONOOH)) and can inactivate various microorganisms (such as viruses, bacteria, and fungi) [16,17,18,19,20,21,22]. Without the limitation of the CAP device, PAW can be used as a carrier of RONS to decontaminate foods in a variety of scenarios [23]. However, the decontamination effects of PAW against *A. flavus* and Aflatoxin are not clear yet.

Herein, this study investigated the immediate fungicidal effect and long-term inhibitory effect of PAW on *A. flavus*. A novel plasma jet device was employed to prepare PAW, and the electrochemical properties (oxidation-reduction potential (ORP), pH, and electrical conductivity) of PAW were detected. Meanwhile, the concentration of ONOO^−^ and NO_3_^−^ in PAW was also measured. Furthermore, the inactivation efficiency of PAW against *A. flavus* at different storage times and its relationship with ONOO^−^ concentration were analyzed. Then, the possible inactivation mechanism of PAW against *A. flavus* was analyzed by measuring the cell membrane integrity, intracellular RONS, and mitochondrial membrane potential. In addition to the inactivation effect, the inhibition effect of PAW on AFB_1_ synthesis was also investigated via the analysis of the changes in antioxidant capacity, energy metabolism, and the expression of key genes in the aflatoxin synthesis pathway of *A. flavus*.

## 2. Experimental Setup and Methods

### 2.1. The Preparation of PAW

**Figure 1:**
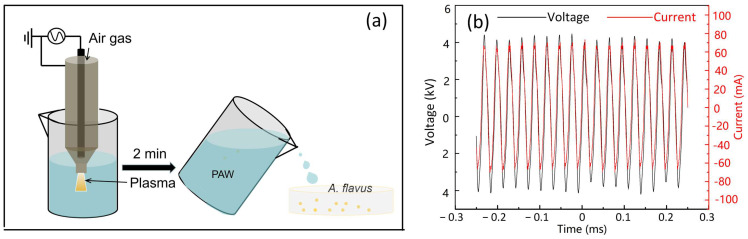
(**a**) Schematic diagram of CAP jet device, PAW preparation, and PAW treatment of *A. flavus*. (**b**) Current and voltage waveforms of CAP jet.

A novel CAP jet device was employed to treat the water for the preparation of PAW (Figure 1a). The plasma jet device was comprised of an HV copper electrode in the chamber and the outer copper electrode as the ground electrode. The electrodes were separated by a ceramic tube and were driven by the high-voltage power supply (Suodifu, Zhengzhou, China). Air was used as the carrier gas with a flow rate of 10 L/min, and the length of the jet plume was about 30 mm. The discharge voltage was measured by a high-voltage probe (Tektronix, P6015A), and the discharge current was measured by the current probe (Pearson Electronics, 2877). The data of the waveforms were recorded by an oscilloscope (Teledyne, WaveSurfer 3000). As shown in Figure 1b, the discharge frequency was 33 kHz and the voltage had a peak-to-peak value of 8 kV. The power was sustained at approximately 240 W. 50 mL sterile distilled water was treated by CAP for 2 min to acquire the PAW solution. After being stored for different times, PAW was utilized to treat *A. flavus.*

### 2.2. Measurements of Optical Emission Spectra

During the processing of CAP discharge, optical emission spectra (OES) were detected by AvaSpec-ULS 4096CL-EVO (Avantes, Lafayette, CO, USA). The probe of fiber optics cable was used to acquire the light signals at a distance approximately 5 mm away from the end of the plasma jet plume.

### 2.3. Measurements of pH, ORP, Electrical Conductivity, and ONOO^−^ in PAW

The pH and ORP of PAW were measured after storage at different times by the multimeter pH and Redox (Mettler-Toledo, Zurich, Switzerland). The electrical conductivity of PAW was measured after storage at different times by an electric conductivity meter. The ONOO^−^ concentration in PAW was measured after storage for different times by the method provided by Tarabová et al. [24]. The probe of 2, 7-dichlorodihydrofluorescein diacetate (H_2_DCFDA) was used for ONOO^−^ detection. NO_3_^−^ concentration in PAW was measured after storage for different times by spectrophotometric method [25]. 

### 2.4. The Culture of A. flavus Spores and Mycelium

*A. flavus* spores were harvested in 20 mL of sterile deionized water from 7-day-old cultures grown on potato dextrose agar (PDA) at 30 °C. After filtration with the sterile gauze, centrifuged, and washed with the sterile deionized water, the concentration of *A. flavus* spores was adjusted to about 7 log CFU/mL for PAW treatment. In addition, *A. flavus* spores were inoculated on PDA and cultivated for two days at 30 °C. Then, the mycelium was collected and treated by PAW. 

### 2.5. Detection of A. flavus Inactivation Efficiency

The inactivation efficiency of *A. flavus* by PAW was evaluated by colony forming units (CFU). *A. flavus* spores were treated by PAW after storage at different times, and then centrifuged at 8000× *g* for 5 min and resuspended by the sterile deionized water. A 10-fold serial dilution of 100 μL of spore suspensions were spread on PDA. Then the plates were placed in the incubator for the spore growth at 30 °C. After 48 h cultivation, the CFU was counted.

### 2.6. Detection of Cell Membrane Integrity, Intracellular RNS and Mitochondrial Membrane Potential 

After treatment with the instant-prepared PAW, the cell membrane integrity, intracellular RNS, and mitochondrial membrane potential of *A. flavus* were measured by flow cytometry and fluorescence microscope. Propidium iodide (PI) was used to detect the cell membrane permeability. 2′, 7′-dichlorofluorescein diacetate (H_2_DCF-DA), 3-Amino,4-aminomethyl-2′,7′-fluorescein, and diacetate (DAF-FM DA) were used to detect the intracellular ONOO^−^ and NO. 5,5′,6,6′-tetrachloro-1,10,3,3′-tetraethylimidacarbocyanine iodide (JC-1) (Beyotime, Shanghai, China) was used to detect the mitochondrial membrane potential (MMP). After PAW treatment, *A. flavus* spores or mycelium were centrifuged and resuspended by phosphate-buffered saline (PBS), and their probes were added into the suspensions and incubated at 30 °C for 30 min in the dark. Then, *A. flavus* spores or mycelium were washed by PBS, and the mean fluorescence intensity (MFI) of *A. flavus* spores was analyzed by flow cytometry. The fluorescence intensity of mycelium was observed by fluorescence microscope.

### 2.7. Determination of AFB_1_ Concentration

AFB_1_ was detected with enzyme-linked immunosorbent assay (ELISA). After treatment with the instant-prepared PAW, *A. flavus* was inoculated onto PDA and cultivated for 2, 3, and 5 d. The AFB_1_ concentrations of *A. flavus* at different culture times were analyzed by the AFB_1_ ELISA Kit (Finderbio, Shenzhen, China). The kit consisted of a 96-well microtiter plate pre-coated with conjugate antigen, horseradish enzyme marker, antibody, standard of AFB_1_, and other supporting reagents. AFB_1_ in the samples can compete with conjugate antigen pre-coated in the plate, and inhibit AFB_1_ antibodies. TMB (3,30,5,50-tetramethylbenzidine) was used for color development. The absorbance value of the sample was negatively correlated with the content of AFB_1_. The amount of AFB_1_ in the samples can be calculated by comparing it with the standard curve. The detection limit of this ELISA kit was 1 ppb.

### 2.8. Determination of SOD Activity, CAT Activity, and ATP Content

After treatment with the instant-prepared PAW, *A. flavus* was inoculated onto PDA and cultivated for 2, 3, and 5 d. The SOD activity, CAT activity, and ATP content of *A. flavus* at different culture times were measured. Mycelia were freeze-dried in liquid nitrogen and grounded. After that, the samples were homogenized in PBS and centrifuged in 7000× *g* for 15 min at 4 °C. The supernatant was used for the detection of ATP content, total protein concentration, and enzyme activity. The ATP level was measured with a bioluminescence assay kit (Beyotime, Shanghai, China). The total protein concentration of *A. flavus* was determined by Bradford assay (Protein standard: bovine serum albumin). SOD and CAT activities were detected by commercial kits (Cu/Zn-SOD and Mn-SOD Assay Kit, Catalase Assay Kit, Beyotime, Shanghai, China). The SOD or CAT activities were expressed as the SOD activity or CAT activity divided by total protein concentration.

### 2.9. Detection of the Gene Expression Level

After treatment with the instant-prepared PAW, *A. flavus* was inoculated onto PDA and cultivated for 2, 3, and 5 days for the extraction of RNA. The cultures were harvested and homogenized in liquid nitrogen. Total RNA was extracted with TRIzol Reagent (Thermofisher Scientific, Carlsbad, USA) and transcribed to cDNA by the *Evo M-MLV* RT premix Kit (Accurate Biology, Changsha, China) according to the manufacturer’s protocol. The relative expression of aflatoxin biosynthesis genes (*aflD*, *aflP*, *aflQ*, *aflR*, *aflS*) was determined by real-time fluorescence quantitative PCR (RT-qPCR). The primers for the amplification of these 5 genes and a reference gene (β-tubulin) were reported by the literature [26]. The sequences and amplification efficiency of each pair of primers were also shown in the Supporting Information (Table S1 and Figure S1). The PCR reaction contained cDNA as a template, gene-specific primers, and 2 × SYBR^@^ Green *Pro Taq* HS Premix (Accurate Biology, Changsha, China). qPCR was carried out at 95 °C for 2 min; 40 cycles of 95 °C for 15 s, 58 °C for 15 s, and 68 °C for 20 s (Roche’s automatic fluorescence PCR analyzer (LightCycler480)); melting curves were performed according to the default parameters of the instrument. The 2^−∆∆Ct^ values were obtained using the *β-tubulin* gene as an internal reference [27].

### 2.10. Statistical Analysis

Three replicate experiments were carried out to obtain the data. Values were expressed as the mean value ± standard deviation (SD). Analysis of variance (ANOVA) in SPSS statistical package 22.0 (SPSS statistical package 22.0, New York, NY, USA) was used to perform the statistical analysis. Student–Newman–Keuls multiple range test was employed to identify the significant differences between samples with a confidence level at *p* < 0.05.

## 3. Results and Discussion

### 3.1. The OES of Plasma Jet

**Figure 2:**
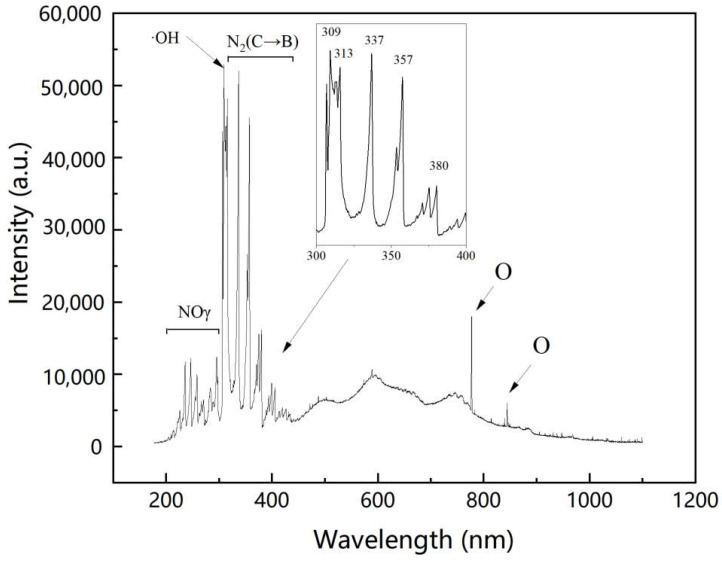
OES of the plasma in the range of 200–1100 nm.

To identify the major excited reactive species, OES in the wavelength of 200–1000 nm were observed. As was shown in Figure 2, the second positive system N_2_ (C^3^Π_u_ → B^3^Π_g_) emissions (313, 337, 357, and 380 nm), NO_γ_ (200–300 nm), O (777 and 844 nm) and ·OH band (309 nm) could be identified [28]. The electron impact dissociation of O_2_ molecules led to the formation of O (Equation (1)). Simultaneously, the band in N_2_ molecules can also be broken by vibrational excitation and dissociation (Equation (2)) [29]. The excited N_2_ molecules can dissociate H_2_O molecules to form ·OH (Equations (3) and (4)). Nitrogen oxides can be formed from the reactions of the dissociated N_2_ and O_2_ (Equations (5) and (6)) [7]. These gas-phase RONS can further react with water molecules to produce various secondary RONS in liquids [17].

*e* + O_2_ → O + O + *e* (1)

*e* + N_2_ → N + N + *e* (2)

*e* + N_2_ → N_2_* + *e* (3)

N_2_* + H_2_O → N_2_ + ·OH + H (4)

N + O → NO(5)

NO + O → NO_2_ (6)

### 3.2. Physicochemical Properties of PAW

**Figure 3:**
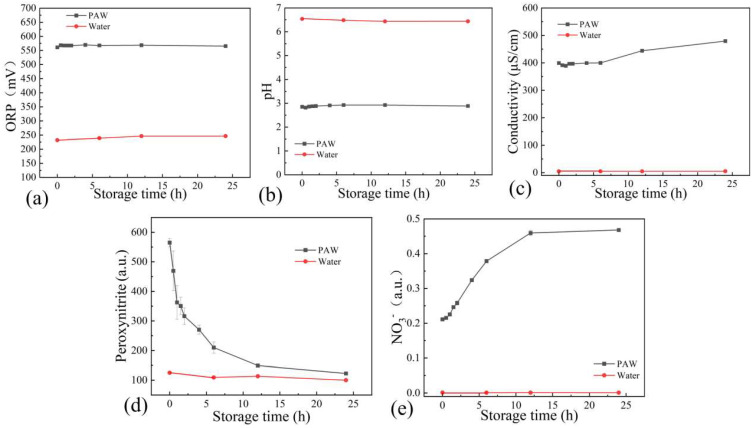
The changes of (**a**) ORP, (**b**) pH, (**c**) electrical conductivity, (**d**) ONOO^−^, and (**e**) NO_3_^−^ in PAW within 24-h storage.

The plasma gas-liquid interaction can change the physicochemical properties of the liquid. ORP can reflect the total oxidation level of the solution. Generally speaking, the higher ORP, the stronger oxidation of the solution. It has been reported that the ORP of water after CAP treatment was increased [20]. Compared to water (~230 mV), the ORP of PAW was increased to about 560 mV (Figure 3a). In addition to ORP, the acidification of water is another property after CAP treatment. As was shown in Figure 3b, the pH value of PAW was about 2.8. The results of OES demonstrated the generation of nitrogen oxides. The dissolution of nitrogen oxides in the liquid can form NO_2_^−^, NO_3_^−^, and H^+^ ions, thus leading to a drop in pH (Equations (7) and (8)) [7]. Electrical conductivity was also measured. As was shown in Figure 3c, the electrical conductivity of PAW was increased to about 400 µS/cm, indicating that a number of ions existed in PAW. These ions, especially RONS, play important roles in fungal inactivation. 

Peroxynitrite was reported to be the critical RONS in PAW, which was a strong oxidant agent including both anionic (ONOO^−^) and protonated (ONOOH) forms [16]. Peroxynitrite can be generated in PAW through many pathways. Firstly, ∙O_2_^−^ reacted with NO, and ·OH reacted with NO_2_ to form ONOO^−^ (Equations (9) and (10)) [30]. Secondly, ONOO^−^ can be formed by the dissolution of gaseous N_2_O_5_ from the plasma region (Equation (11)). In addition, ONOO^−^ can be formed via the reaction of NO_2_^−^ and H_2_O_2_ (Equation (12)) [31]. Peroxynitrite was a kind of short-lived RONS. As was shown in Figure 3d, ONOO^−^ was gradually decreased with the increasing storage time. Given that ONOOH would be the dominant form when the pH was lower than 6.8 (p*K_a_* of ONOOH), ONOOH was the dominant form in PAW, whose pH was 2.8. It was reported that most of ONOOH would be decomposed into NO_3_^−^ and H^+^ (Equation (13)) [16]. Thus, we speculated that peroxynitrite was decomposed via this reaction. The increase in NO_3_^−^ during the extension of storage time could also verify this viewpoint (Figure 3e). The slight increase of electrical conductivity from 400 to 480 µS/cm during 24-h storage may be attributed to the generation of NO_3_^-^ (Figure 3c). While there was no significant change in ORP with the increasing storage time. This may be attributed to the pH, which was an important factor influencing ORP [32].

2NO_2_ + H_2_O → NO_2_^−^ + NO_3_^−^ + 2H^+^ (7)

NO + NO_2_ + H_2_O → 2NO_2_^−^ + 2H^+^ (8)

∙O_2_^−^ + NO → ONOO^−^ (9)

·OH + NO_2_ → ONOO^−^ + H^+^ (10)

N_2_O_5_ + H_2_O → 2ONOOH (11)

NO_2_^−^ + H_2_O_2_ → H_2_O + ONOO^−^ (12)

ONOOH → NO_3_^−^ + H^+^ (13)

### 3.3. The Inactivation of A. flavus by PAW

**Figure 4:**
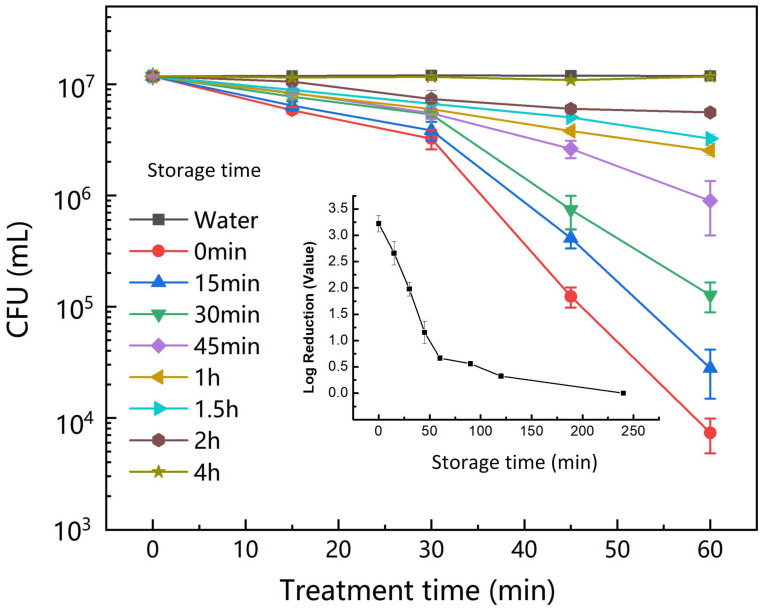
The changes of CFU treated by PAW at different storage times.

Although the fungicidal ability of PAW has been evidenced [18,33], the influence of storage time on the fungicidal efficiency of PAW was rarely studied. As was shown in Figure 4, the instant-prepared PAW exhibited the best fungicidal ability, which achieved a 3.22 Log reduction of *A. flavus* after 60-min treatment. When the PAW was stored at different times, its fungicidal ability was reduced quickly. A 60-min PAW treatment could only reduce 0.7 Log of *A. flavus* after 1-h storage. When the storage time reached 4 h, the fungicidal ability of PAW almost disappeared, which was the same as that of water treatment (Figure 4). According to the detection of peroxynitrite in PAW (Figure 3d), the fungicidal ability of PAW was consistent with the change of peroxynitrite, which indicated that peroxynitrite plays an important role in *A. flavus* inactivation by PAW.

### 3.4. The Antifungal Mechanism of A. flavus by PAW

**Figure 5:**
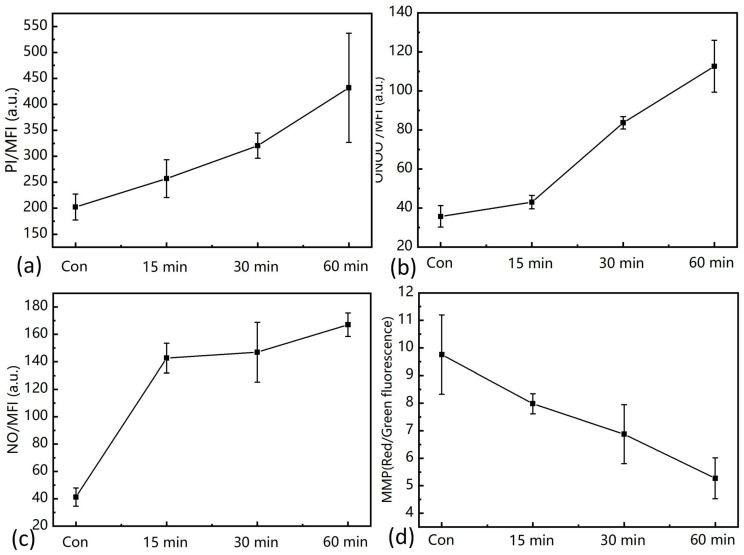
The change of (**a**) cell membrane integrity, (**b**) intracellular ONOO^−^, (**c**) NO, and (**d**) mitochondrial membrane potential after PAW treatment for 15, 30, and 60 min.

It was reported that RONS in PAW could induce membrane oxidative damage [18]. Among RONS, peroxynitrite could lead to lipid peroxidation and nitration [16]. Under the condition of low pH, peroxynitrite mainly in the form of ONOOH could cross the lipid bilayer in the cell membrane, and then initiate membrane peroxidation [34]. As was shown in Figure 5a, the cell membrane integrity of *A. flavus* spores was damaged. The enhancement of the permeability will contribute to the penetration of RONS into the cell, which can damage the cellular components and lead to the death of *A. flavus*. As was shown in Figure 5b,c, with the increase in PAW treatment time, both intracellular ONOO^−^ and NO increased. Differently, after 15 min PAW treatment, the increase in NO was more obvious than ONOO^−^. The reason may be that ONOO^−^ was not easy to enter a cell when the membrane was relatively intact. While, NO was more stable and highly diffusible, which could enter the cells easily [34]. Mitochondria dysfunction is an important intracellular event for the fungal inactivation induced by CAP or PAW [18,35]. As was shown in Figure 5d, PAW induced the depolarization of MMP. NO could lead to the loss of MMP through signal regulation (glutamate-receptor activation) [36]. In addition, NO could regulate ATP synthesis by inhibiting cytochrome c oxidase [37]. ONOO^−^ could inactivate the complex I, II, and V, as well as the electron transport-related enzymes, thus inducing mitochondrial dysfunction [19]. Thus, the increase in NO and ONOO^−^ could lead to a drop in MMP, which resulted in the death of *A. flavus* eventually.

**Figure 6:**
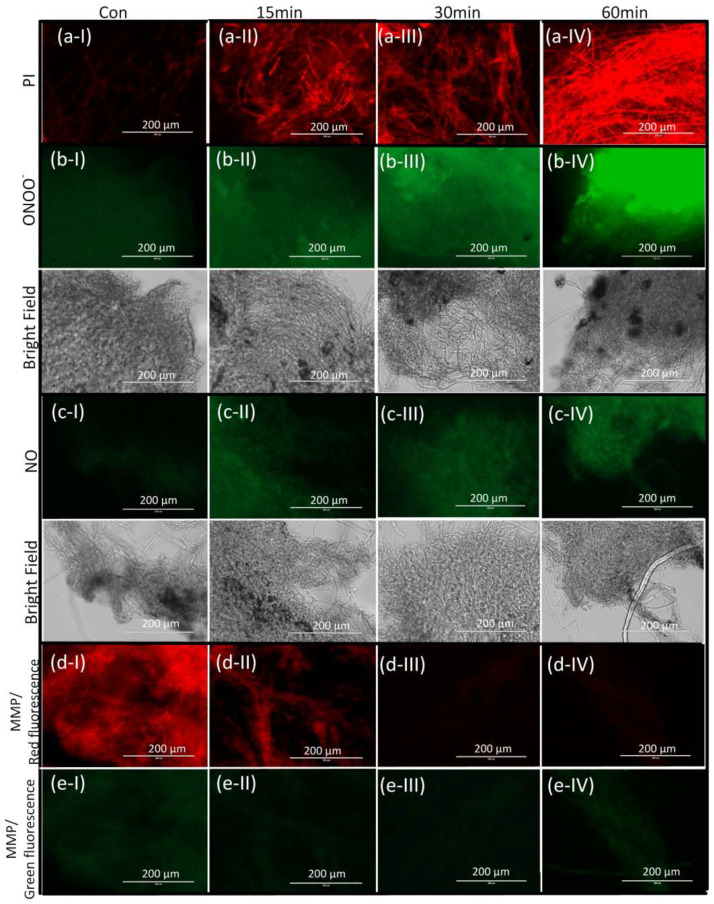
The change of (**a**) cell membrane integrity, (**b**) intracellular ONOO^−^, (**c**) NO, and (**d**,**e**) mitochondrial membrane potential in *A. flavus* mycelium after PAW treatment for 15, 30 and 60 min.

In addition to *A. flavus* spores, the change in cell membrane integrity, intracellular ONOO^−^ and NO, and MMP in *A. flavus* mycelium was also detected. As was shown in Figure 6, PAW treatment could also lead to the increase in cell membrane permeability and intracellular NO and ONOO^−^, as well as the depolarization of MMP. These results indicated that PAW could also inactivate *A. flavus* mycelium. 

### 3.5. The Inhibition of AFB_1_ Synthesis by PAW

**Figure 7:**
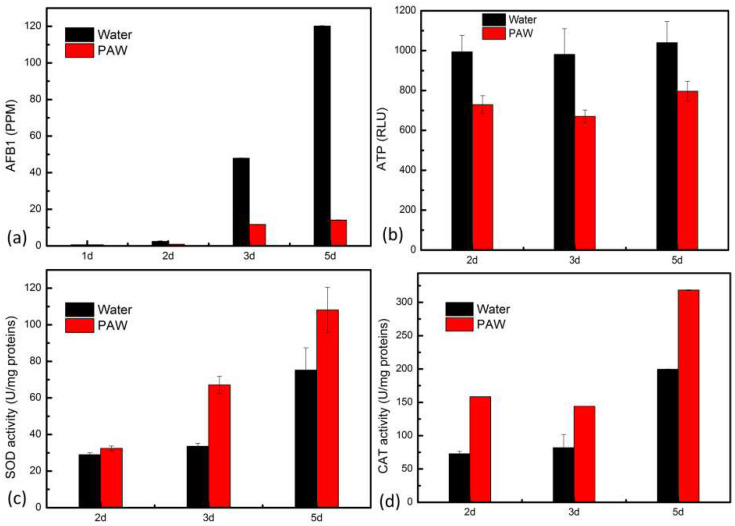
The changes of (**a**) AFB_1_, (**b**) ATP, (**c**) SOD activity, and (**d**) CAT activity during 5-d cultivation before and after PAW treatment.

Besides the fungicidal ability of PAW, the long-term influence of PAW on fungal growth and AFB_1_ synthesis in *A. flavus* was also investigated. After the instant-prepared PAW treatment, the spores were inoculated onto PDA and cultivated for 2, 3, and 5 d. As was shown in Figure 7, the content of AFB_1_ reached 120 ppm after 5-d cultivation in the control group, while the content of AFB_1_ in the PAW treatment group was about 14 ppm. The result indicated that PAW treatment could influence the AFB_1_ synthesis of *A. flavus*. The influence of plasma treatment on intracellular redox homeostasis and energy metabolism was considered the key reason for inducing fungal biological effects [35]. Therefore, we investigated the change of intracellular ATP, SOD, and CAT activity during 5-d cultivation after PAW treatment. It was found that ATP in the PAW treatment group was less than that in the control group. While SOD and CAT activity were higher compared with the control group (Figure 7). Liao et al. reported that the oxidative stress response triggered by plasma treatment will consume more cellular energy [38]. Thus, the energy assigned for other physiological activities might be reduced. It was speculated that the energy assigned for AFB_1_ synthesis might be reduced when *A. flavus* suffered the oxidative stress of PAW treatment, thus leading to the decrease in AFB_1_. In addition, it was reported that plasma treatment could disrupt mitochondrial function and decrease acetyl-CoA contents [39]. Acetyl-CoA is the precursor of Aflatoxins (AFs), which can be cyclized by the polyketide synthase. With a series of enzymatic reactions, AFs can be synthesized [40]. PAW treatment might disrupt the mitochondrial function and decrease acetyl-CoA contents, thus inhibiting the synthesis of AFB_1_. Meanwhile, the enhancement of antioxidant enzymatic activities might inhibit AFB_1_ synthesis. For example, dithiothreitol, and dimethyl sulfoxide can inhibit AFB_1_ synthesis with an increase in SOD enzymatic activity [41,42]. Similarly, it was reported that ascorbic acid, cinnamaldehyde, and piperine-induced AFB_1_ decrease with an increase in CAT activity [42,43,44]. Therefore, the improvement of SOD and CAT activities might inhibit AFB_1_ synthesis under PAW treatment. 

### 3.6. Inhibition of Key Genes Expression of AFB_1_ Synthesis in A. flavus by PAW

**Figure 8:**
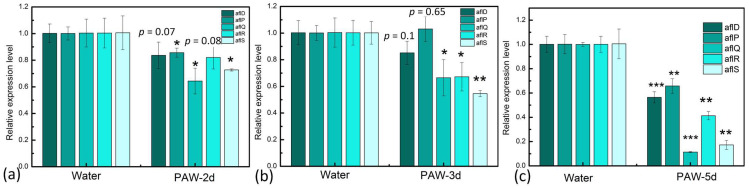
The relative expression levels of *aflD*, *aflP*, *aflQ*, *aflR* and *aflS* genes involved in the synthesis of AFB_1_ during 2-d (**a**), 3-d (**b**) and 5-d (**c**) cultivation before and after PAW treatment. Significantly different values are expressed as * *p* < 0.05, ** *p* < 0.01, *** *p* < 0.0001.

In *A. flavus*, the biosynthesis pathways of AFB_1_ are organized by diverse regulatory and structural genes. About 25 genes residing in a 75 kb cluster can encode more than 18 enzymes [45]. The structural genes of *aflD, AflP,* and *aflQ* encode three kinds of key enzymes in the biosynthesis pathways of AFB_1_. *AflD*-encoded enzyme is responsible for the conversion of norsolorinic acid (NOR) to averantin (AVN). *AflP*-encoded enzymes are necessary for converting sterigmatocystin (ST) to o-methylsterigmatocystin (OMST). And *aflQ*-encoded enzymes are necessary for converting OMST to AFB_1_. The relative expression of these three structural genes was examined. As was shown in Figure 8, all of these three genes were downregulated during 5d cultivation. *aflR* and *aflS* are key regulatory genes in the synthesis of AFB_1_ [26]. We also examined the relative expression of these two genes. As was shown in Figure 8, the expression of these two genes was also downregulated during 5-d cultivation. It was speculated that the changes in intracellular redox homeostasis and energy metabolism induced by PAW could regulate the expression of genes involved in the AFB_1_ synthesis pathway, consequently decreasing AFB_1_ biosynthesis in *A. flavus*.

## 4. Conclusions

In this study, the immediate fungicidal effect and long-term inhibitory effect of PAW on *A. flavus* were investigated. PAW could efficiently inactivate *A. flavus*, which was attributed to the increase in cell membrane permeability and intracellular NO and ONOO^−^ as well as the depolarization of mitochondrial membrane potential. The fungicidal ability of PAW almost disappeared after 4-h storage. Besides the immediate fungicidal effect, PAW also exhibited long-term fungistatic properties. PAW treatment could inhibit AFB_1_ production by inhibiting the expression of key genes involved in AFB_1_ biosynthesis. Peroxynitrite was considered to play a critical role in the inactivation and inhibition of *A. flavus.* This study could provide new guidance for the application of low-temperature plasma technology especially PAW in the fungal and mycotoxin decontamination.

## Data Availability

Data will be made available on request.

## Supplementary Materials

The following supporting information can be downloaded at: https://www.mdpi.com/article/10.3390/foods12132490/s1, Table S1: The list of primers and the corresponding clustered aflatoxin biosynthesis pathway genes showing enzymes involved and their functions in the study; Figure S1: Representative standard curves and efficiency of each pair of primers. (a) β-Tubulin, (b) aflD, (c) aflP, (d) aflQ, (e) aflR, and (f) aflS. (Solid red lines denote the theoretical calculation curves. Dashed black lines indicate measurement curves).
