# Supplementary material for: Inhibition of Fungal Growth and Aflatoxin B1 Synthesis in Aspergillus flavus by Plasma-Activated Water
Source: Foods. 2023 Jun 26;12(13):2490. doi: 10.3390/foods12132490 (PMC10340411; doi:10.3390/foods12132490)
Supplement: Supplementary file 1 [file foods-12-02490-s001.zip › foods-2401454-supplementary.pdf]

Supporting Information

Inhibition of fungal growth and aflatoxin B1 synthesis in *Aspergillus flavus* by plasma-activated water

Yao Qihuan[1,2,3,#], Xu Hangbo[1,2,3,#], Zhuang Jie[4], Cui Dongjie[1,2,3,*], Ma Ruonan[1,2,3,*] and Jiao Zhen[1,2,3]

1 Zhengzhou Research Base, State Key Laboratory of Cotton Biology, School of Agricultural Sciences, Zhengzhou University, Zhengzhou 450001, China

2 Henan Key Laboratory of Ion-Beam Bioengineering, School of Agricultural Sciences, Zhengzhou University, Zhengzhou 450052, China

3 Sanya Institute, Zhengzhou University, Zhengzhou 450001, China

4 Suzhou Institute of Biomedical Engineering and Technology, Chinese Academy of Sciences, Suzhou 215163, China

* Corresponding authors.

E-mail addresses: cuidongjie0323@zzu.edu.cn (D. Cui) and maruonan@zzu.edu.cn (R. Ma).

# These authors contributed equally to this work.

Table S1. The list of primers and the corresponding clustered aflatoxin biosynthesis pathway genes showing enzymes involved and their functions in the study.

Target Gene	Sequences (5'—3')	Function in the Biosynthetic Pathway	Target Size (bp)
β-Tubulin	Forward:CTTGTTGACCAGGTTGTGGAT<br>Reverse: GTCGCAGCCCTCAGCCT	Reference housekeeping gene	51
aflD	Forward: GTCCAAGCAACAGGCCAAGT<br>Reverse: TCGTGCATGTTGGTGATGGT	Norsolorinic acid (NOR) → averantin (AVN)	66
aflP	Forward: GGCCGCCGCTTTGATCTAGG<br>Reverse: ACCACGACCGCCGCC	Sterigmatocystin (ST) → O-methylsterigmatocystin (OMST)	123
aflQ	Forward: GTGTCCGCAGTGTCTAGCTT<br>Reverse: GCTCAAAGGTCGCCAGAGTA	O-methylsterigmatocystin (OMST) → aflatoxin B1(AFB1)	115
aflR	Forward: CTCAAGGTGCTGGCATGGTA<br>Reverse: CAGCTGCCACTGTTGGTTTC	Pathway regulator	86
aflS	Forward: CTGCAGCTATATTGCCCACA<br>Reverse: TAAACCCAGGCAGAGTTGGT	Pathway regulator	117

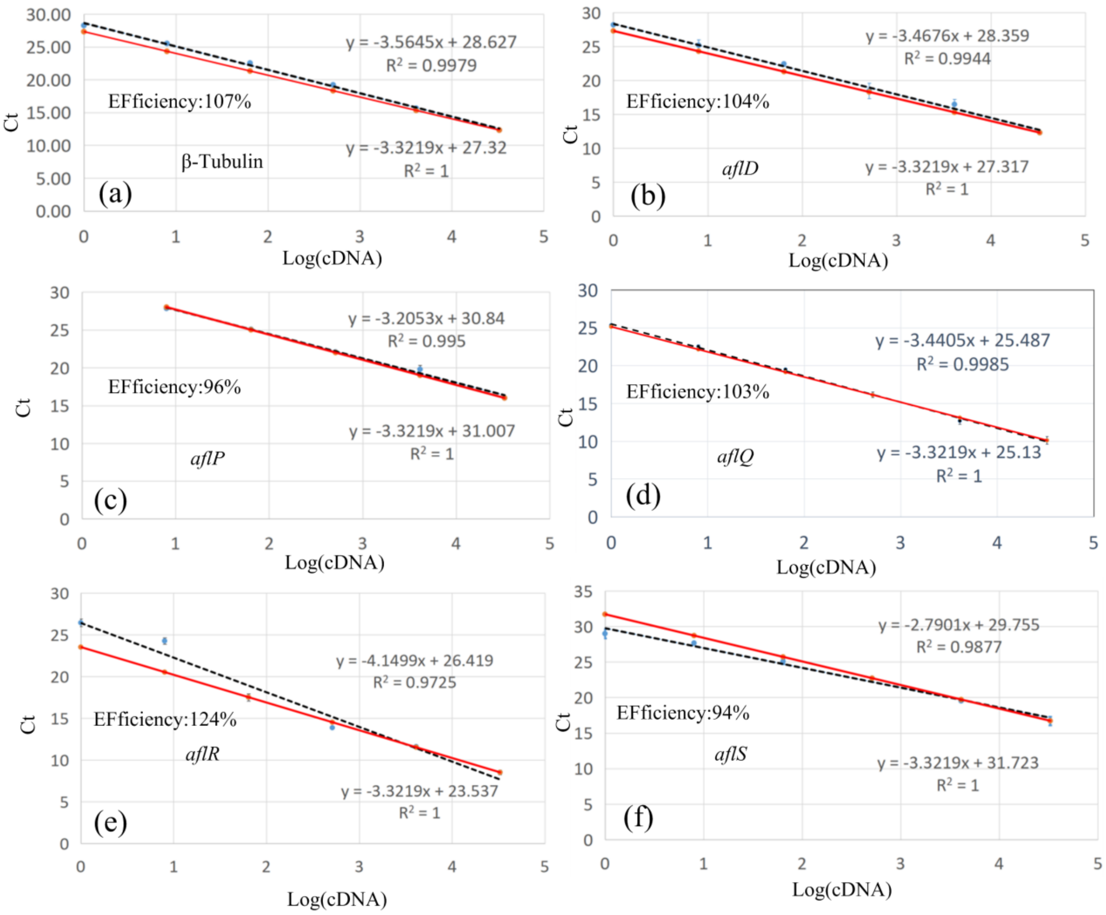

Figure S1: Representative standard curves and efficiency of each pair of primers. (a) *β-Tubulin*, (b) *aflD*, (c) *aflP*, (d) *aflQ*, (e) *aflR*, and (f) *aflS*. (Solid red lines denote the theoretical calculation curves. Dashed black lines indicate measurement curves)
